# Mechanisms underlying interactions between PAMAM dendron-grafted surfaces with DPPC membranes†

**DOI:** 10.1039/c8ra03742f

**Published:** 2018-07-11

**Authors:** Jia Li, Kai Jin, Srinivas C. Mushnoori, Meenakshi Dutt

**Affiliations:** Department of Chemical and Biochemical Engineering, Rutgers, The State University of New Jersey Piscataway New Jersey 08854 USA meenakshi.dutt@rutgers.edu

## Abstract

Biofouling is a pervasive problem which demands the creation of smart, antifouling surfaces. Towards this end, we examine the interactions between a dipalmitoylphosphatidylcholine (DPPC) lipid bilayer and a polyamidoamine (PAMAM) dendron-grafted surface. In addition, we investigate the impact of dendron generation on the system behavior. To resolve the multiscale dynamical processes occurring over a large spatial scale, we employ Molecular Dynamics simulations with a coarse-grained implicit solvent force field. Our results demonstrate the transient and equilibrium system dynamics to be determined by the PAMAM dendron generation along with the underlying mechanisms. Higher generation dendrons are observed to favor penetration of the DPPC molecules into the dendron branches, thereby enabling sustained interactions between the membrane and the dendron-grafted surface. Under equilibrium, the membrane adopts a bowl-shaped morphology whose dimensions are determined by the dendron generation and density of interactions. The results from our study can be used to guide the design of novel surfaces with selective antifouling properties which can prevent the adsorption of microorganisms onto lipid membranes.

## Abstract 2

The interactions between a DPPC lipid membrane and a PAMAM dendron-grafted surface.

## Graphical abstract

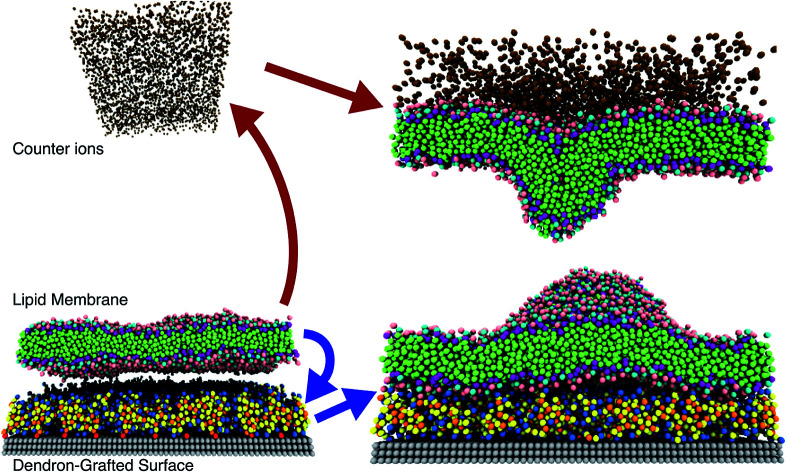

## Introduction

Biofouling presents risks in a wide range of situations including the industrial production of medical devices,^1–3^ food packaging and storage safety,^4–7^ and the operation of medical implants and under water equipment.^8–12^ Hence, interest in the development of new antifouling materials and coatings which deter the interfacial adsorption of proteins, bacteria or other organisms onto surfaces has continued to surge during the past decades.^9,13^ Experimental studies have shown that polymer brushes grafted onto supporting substrates are well suited for the purpose.^14–19^ For example, poly(amidoamine) (PAMAM)-based dendritic surfactant polymers have been successful as antifouling materials by diminishing the adhesion of platelets onto a hydrophobic substrate by over 90%.^20^ The dendritic polymers were adsorbed onto the substrate. Both experimental^21–25^ and computational^26–31^ studies have demonstrated PAMAM dendrimers^32,33^ to interact with and penetrate lipid membranes. Since a significant fraction of microorganisms encompass lipid bilayers, these findings highlight the potential of PAMAM to serve as an antifouling material. Molecular Dynamics simulations used in conjunction with a coarse-grained force field^34,35^ demonstrated PAMAM dendrimers to induce pore formation in lipid bilayers.^26,27^ The outcome of this study was dependent upon the concentration and size of the dendrimers. Using a similar approach, the protonation level of PAMAM dendrimers was also determined to influence their interactions with cell membranes.^28^ Where as PAMAM dendrons with carboxylate terminal groups have been grafted onto silica substrate to protect it against the adsorption of heavy metal ions,^36^ similar surfaces have yet to be explored for their antifouling properties. In addition, the interactions between PAMAM dendron-grafted surface and lipid bilayers has not been investigated.

In this study, we examine the mechanisms underlying the interactions between lipid bilayer membranes and PAMAM dendron-grafted surfaces and probe the role of dendron generation. To obtain multiscale resolution of the dynamical processes across large spatial scales, the study was performed using Molecular Dynamics simulations along with a coarse-grained implicit solvent force field. We find the mechanisms underlying the interactions between the PAMAM dendron-grafted surface and the lipid membrane to be dictated by the dendron generation. The results from our study can guide the design of substrates encompassing grafted hyper-branched polyelectrolytes with selective antifouling properties.

## Methodology

The particle dynamics was resolved by using classical molecular dynamics (MD) simulations.^37–39^ MD generates the trajectories of particles by numerically integrating Newton's equations of motion. The forces acting on the beads can be expressed as the gradients of the total potential energy, which encompasses contributions from pair, bond and angle interactions. The velocity Verlet method was used to integrate the equations of motion because of its greater stability, time reversibility and better preservation of the symplectic form in the phase space over the Euler method.^39^ The MD simulations were run using the open source parallelized MD program named LAMMPS.^40^

We investigated the mechanisms underlying the interactions between a lipid bilayer membrane and a dendron-grafted surface. To understand the impact of the interactions on the molecular and material properties of the bilayer, we simulated a 29.7 nm × 29.7 nm lipid bilayer. The membrane dimension was selected based upon several considerations. These considerations include the dimensions of the membrane being sufficiently large to enable the simultaneous interaction of the membrane with several dendrons of generation G5 which have been grafted at points which are uniformly separated from each other; the ability of the membrane to potentially bend due to excess area induced *via* interactions with the dendrons or counter ions; the total area of the membrane being several orders of magnitude larger than the area per lipid, and the membrane initially being in a tensionless state. For computational efficiency, the bilayer and the surface were represented through an implicit solvent coarse-grained (CG) model which enables the resolution of larger spatial scales by reducing the number of degrees of freedom.

The lipid bilayer encompassed 3200 dipalmitoylphosphatidylcholine, or 1,2-dipalmitoyl-*sn*-glycero-3-phophocholine, (DPPC) molecules.^34,35,41^ DPPC is a common lung surfactant which is frequently used to model cell membranes.^42–46^ Each lipid molecule had a hydrophilic head group composed of four CG beads and two hydrophobic tails composed of four beads each. Two of the head beads, the choline and the phosphate groups, had opposite charges (+1*e* and −1*e*). All parameters for the potentials for bonds, angles and non-bonded pairs were taken from the dry MARTINI model.^47^

The PAMAM dendron-grafted surface was modeled at neutral pH.^36,48^ The CG PAMAM dendron was composed of internal tertiary amine junctions, amide branches, terminal protonated primary amines (+1*e*) and a short hydrocarbon chain which connected the dendron to the supporting surface.^49^ Hydrated chloride counter ions (−1*e*) were added to maintain the charge neutrality of the system. The dendrons were tethered to a surface composed of uniformly distributed hexagonally close-packed amorphous silica beads. The PAMAM dendron-grafted surface spanned 28.2 nm × 28.2 nm in the *x*–*y* plane.

We used the dry MARTINI implicit solvent CG force field^47^ which is based upon the MARTINI model.^34,35^ Four heavy atoms were represented by a single CG bead. The beads were classified into four main categories: polar (P), nonpolar (N), apolar (C) and charged (Q). Under each main category, there were sub-categories which distinguish the beads by their hydrogen-bonding capacities (Q and N) or degrees of polarity (P and C).

The van der Waals component of the non-bonded interactions were modeled by the Lennard-Jones (LJ) potential: 
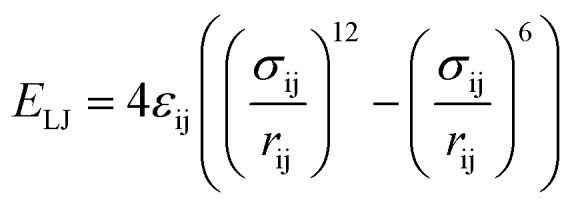
. The parameters *ε*_ij_ were determined by the categories of the interacting beads and set the interaction strength. *σ*_ij_ was the effective bead diameter. The interaction matrix of the explicit solvent MARTINI model was recalibrated in the dry MARTINI force field to capture the strength of the LJ potentials without the solvent. The electrostatic component of the non-bonded interactions between the charged head groups of the DPPC lipids and the protonated amines was modeled by the coulombic potential: 
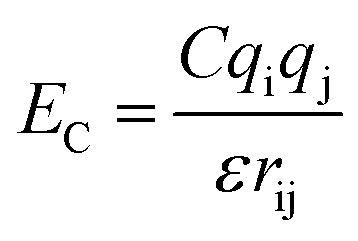
, where *q*_i_ and *q*_j_ were charges carried by beads i and j respectively. The relative permittivity was set to 15. Both the non-bonded interaction potentials have cutoffs of 1.2 nm. The LJ potential was shifted smoothly to zero from 0.9 nm to 1.2 nm. The coulombic potential was shifted smoothly to zero from 0 to 1.2 nm. The neighbor list for each bead (excluding the silica beads) was extended to 1.5 nm and built every time step. The silica surface beads were maintained frozen during the simulations. The bonds were represented by harmonic potential *E*_b_ = *K*_b_(*r* − *r*_0_)^2^. The LJ and coulombic interactions of the bonded atoms were turned off. The angles were represented through the cosine squared potential *E*_a_ = *K*_a_[cos(*θ*) − cos(*θ*_0_)]^2^. The parameters were based upon those used in earlier studies.^34–36,41,48^

A typical cell membrane can have large spatial variations in the bilayer tension.^50–52^ To focus the investigation, we examine the interactions between a tensionless DPPC lipid bilayer with a PAMAM dendron-grafted surface. The free-standing lipid bilayer membrane was equilibrated in the canonical ensemble for 100 ns at 330 K. In order to generate a tensionless membrane, the *x* and *y* dimensions of the simulation box were varied simultaneously (with corresponding changes to the height of the box to maintain the volume constant). The membrane tension *γ* was measured by *γ* = 〈*L*_*z*_ × (*P*_*zz*_ − 1/2*P*_*xx*_ − 1/2*P*_*yy*_)〉,^53^ where *L*_*z*_ is the *z* dimension of the simulation box, *P*_*zz*_ is the normal component of the pressure tensor, and *P*_*xx*_ and *P*_*yy*_ denote the tangential components of the pressure tensor. We obtained an approximately tensionless membrane whose dimensions were 29.7 nm by 29.7 nm.

The PAMAM dendrons were grafted onto an amorphous silica surface so that the neighboring dendron were evenly spaced from each other. The counter ions were distributed uniformly on top of the dendritic brushes to balance the charges and then moved near the upper boundary of the simulation box. The simulation box was set to be 28.2 nm × 28.2 nm in the *x* and *y* dimensions, respectively. The dendron-grafted system was equilibrated in the canonical ensemble for 100 ns. The terminal groups of PAMAM dendrons were protonated primary amines at neutral pH. Due to the highly branched architecture of the dendrons, the crown of each dendron has a non-significant projection over the silica support. The surface coverage was determined by the ratio of the projected area of all dendrons to the total area of the silica surface. When the surface coverage of the PAMAM dendrons over the support was close to full coverage, the dendritic brushes were approximately uniformly distributed over the entire silica support. This allowed the membrane to interact with a homogenous PAMAM-grafted surface, with no vacancies to expose the silica surface to the bilayer. We altered the grafting density for each dendron generation to obtain a fully covered dendron-grafted surface. The details of the PAMAM dendron-grafted surfaces is provided in Table 1.

**Table 1:** Details of PAMAM dendron-grafted surfaces of G1 through G5

Dendron generation	Number of dendron molecules	Number of protonated terminal amines	Surface coverage
1	900	3600	0.98
2	400	3200	0.96
3	225	3600	0.97
4	100	3200	0.92
5	100	6400	1.00

The DPPC membrane and the PAMAM dendron-grafted surface were placed in a simulation box of dimensions 28.2 nm by 28.2 nm in the *x*–*y* plane. The edges of the isolated membrane were trimmed of 454 lipid molecules so that the *x*- and *y*- dimensions of the bilayer and dendron-grafted surface are identical. The total area of the DPPC bilayer was selected such that we would be able to capture the bilayer bending due to interactions with the dendron-grafted surface. The PAMAM dendron-grafted surface was located at the minimum of the *z*-dimension of the simulation box with the DPPC membrane placed above it. The *z* coordinate of the highest dendron bead was the same as the lowest lipid bead to ensure portions of the bilayer and the dendron-grafted surface were within interaction range. The interactions between the bilayer and the dendron-grafted surface would be induced by the van der Waals and electrostatic interactions between the DPPC lipids and the PAMAM dendrons. Since the interactions are activated processes,^54^ this set up was chosen to observe the interactions and the accompanying processes during an interval of time that was computationally feasible. The *z* dimension of the simulation box varied from 66.8 nm to 133.7 nm to maintain a counter ion concentration of 0.1 M. The charges carried by the terminal protonated amine groups were balanced by the chloride ions to preserve the charge neutrality of the system. Since the number of counter ions varied with dendron generation and grafting density, the height of simulation box was altered to ensure that the system remained charge neutral and at a constant counter ion concentration. To focus the investigations on the interactions between the DPPC bilayer and the PAMAM dendron-grafted surface, all the counter ions were frozen near the upper boundary of the simulation box. The counter ions were outside the interaction range from both the membrane and dendron-grafted surface. The boundaries were periodic in the *x* and *y* dimensions, but not in the *z* dimension. The top of the simulation box had a fixed Weeks–Chandler–Anderson wall with *ε* = 3.4 kJ mol^−1^, *σ* = 0.47 nm, and a cut-off distance of 0.528 nm.

**Fig. 1:**
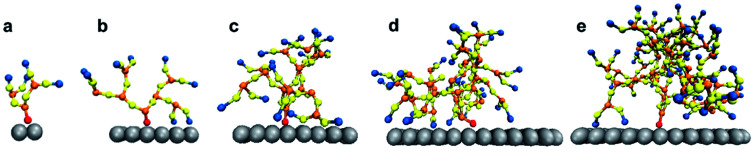
Simulation snapshot of isolated PAMAM dendron of generations (a) G1, (b) G2, (c) G3, (d) G4 and (e) G5. Silica (silver), terminal amine (blue), amide (yellow), internal amine (orange) and hydrocarbon (red) groups are illustrated.

The system was simulated for a time interval which encompasses both the transient and equilibrium phases (∼100 ns) using the canonical ensemble. The transient phase spans the first few nanoseconds of dynamics, followed by the equilibrium phase. The temperature of the system was kept at 330 K through the Berendsen thermostat.^55^ At this temperature, the DPPC lipid membrane would be in the liquid phase so that the individual DPPC molecules have high mobility in the bilayer plane.^56^ The phase of the bilayer is crucial for capturing the interactions between the PAMAM dendron and DPPC lipids. According to the study conducted by Mecke *et al.*,^25^ PAMAM dendrimers interacted selectively with the liquid-crystalline fluid phase bilayers and not with the gel phase bilayers. All simulations were performed using a time step of 10 fs and sampled every 0.1 ns. All the results were obtained using ten independent particle trajectories for each dendron generation. The simulations were repeated for PAMAM dendron generations 1 through 5 (that is, G1, G2, G3, G4 and G5). Fig. 1 shows single dendrons of different generations grafted to a silica surface.

To determine the approximate size of the individual dendrons, we calculated the radius of gyration (*R*_g_) using the following equation: 
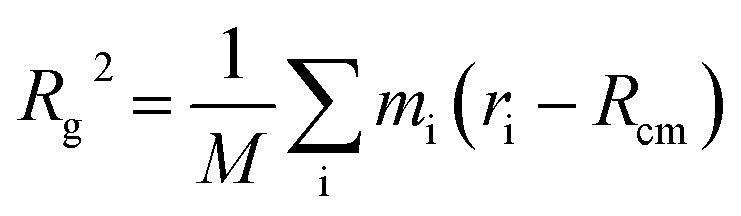
. *M* is the total mass of the dendron molecule, *R*_cm_ is the center-of-mass position, *m*_i_ and *r*_i_ are the mass and position of a dendron bead. During the span of the simulations, the value of the radius of gyration for each dendron generation had minor fluctuations with time. Hence, the size of the PAMAM dendritic brushes were not affected by the interactions with the DPPC membrane.

## Results and discussion

**Fig. 2:**
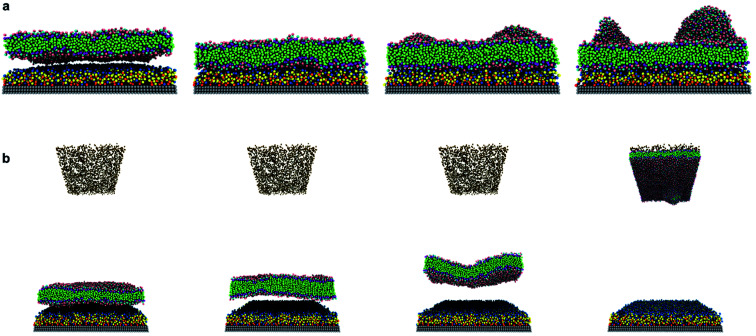
Simulation snapshots of two outcomes of interactions between tensionless DPPC lipid membrane and PAMAM-dendron-grafted surface: (a) membrane develops sustained interactions with PAMAM dendrons; (b) membrane develops sustained interactions with counter ions, at 0 ns, 5 ns, 15 ns and 100 ns from left to right respectively. Choline (pink), phosphate (cyan), glycerol (purple) and hydrocarbon tail (green) groups of DPPC along with terminal amines (blue), amide branches (yellow), internal amines (orange) and tethering hydrocarbon (red) groups of the PAMAM dendron and counter ions (brown) are illustrated.

Our investigations on the interactions between a tensionless DPPC bilayer and PAMAM dendron-grafted surface^36^ yielded two distinct outcomes. In one outcome, the DPPC membrane diffused towards the PAMAM dendron-grafted surface to develop interactions that spanned the remaining simulation interval (Fig. 2(a)) (that is, sustained interactions). In the second outcome, the membrane diffused away from the dendron-grafted surface and moved upwards. The membrane eventually encountered the counter ions and developed sustained interactions with them (Fig. 2(b)). The probability of the two outcomes for the different dendron generations is summarized in ESI Fig. SI1.† For surfaces grafted with lower generations of PAMAM dendrons (G1 through G3), both outcomes were equally likely. With increasing generations, the membrane had a greater tendency to develop long-term interactions with the dendron-grafted surface. After developing sustained interactions with either the dendrons or the counter ions, we observed the membrane to gradually bend to form a bowl-shaped configuration. The membrane sustained this configuration while maintaining its structural stability.

The behavior of the system can be classified into two phases: transient and equilibrium phases. The transient phase encompasses the initial dynamics of the membrane leading to its movement towards the dendrons or the counter ions. The equilibrium phase includes the dynamics of the membrane during its sustained interactions with the dendrons or counter ions, and its bending to form a bowl-shaped configuration. Given the similarity in the results for systems with generations G1 through G3 and G4 through G5, we will be discussing the details of the systems with PAMAM G1 and G5 dendrons.

### Transient phase

For systems where the membrane developed sustained interactions with the dendrons, the membrane was observed to initially diffuse towards the dendrons. The downwards diffusion activated interactions between the choline moiety of the head groups of the membrane lipids and the terminal protonated amines of the dendrons. These interactions coupled with the diffusive motion of the membrane and thermal fluctuations at the molecular scale caused some of the lipids molecules to further penetrate the dendrons. This penetration lead to two types of interactions. The first one was between the choline and phosphate moieties of the lipid head groups and the amide groups of the dendrons. The second interaction was between the glycerol groups of the lipids and the terminal protonated amines of the dendrons. The lipid head groups were observed to have insignificant interactions with the dendron internal tertiary amine junctions and the short hydrocarbon chain grafting the dendron to the silica surface. The hydrocarbon tails of the phospholipids had some interactions with the PAMAM terminal protonated amines. However, most of the interactions occurred between the choline and phosphate moieties of the lipid head groups, and the dendron terminal amines. We surmise that the branched architecture of the PAMAM dendrons served as a barrier to further penetration by the DPPC lipids into the individual dendrons.

**Fig. 3:**
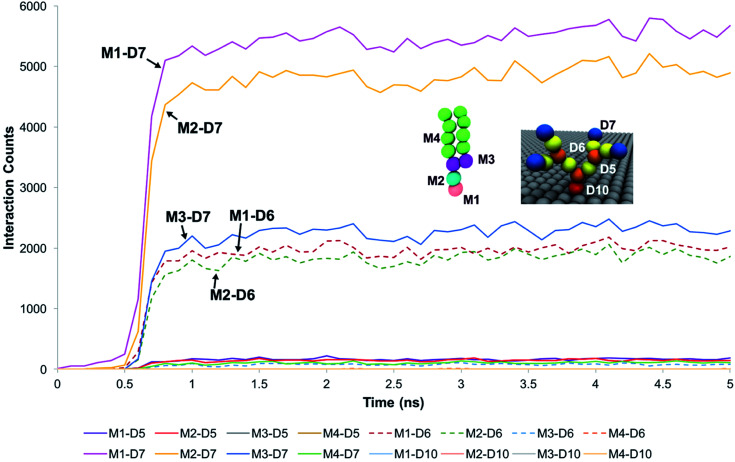
Interaction count measurements between the different bead types of DPPC and the G1 PAMAM dendron when the membrane developed sustained interactions with the dendrons. Choline (pink), phosphate (cyan), glycerol (purple) and hydrocarbon tail (green) groups of DPPC are labeled respectively as M1, M2, M3 and M4. Terminal amines (blue), amide branches (yellow), internal amines (orange) and tethering hydrocarbon (red) groups of the PAMAM dendron are labeled respectively as D7, D6, D5 and D10. Measurements for the first 5 ns is shown as the values remain unchanged from 2 ns onwards.

These observations were supported by measurements of the interaction counts between the distinct bead types encompassing the DPPC membrane and the PAMAM dendrons. Two beads were determined to be interacting with each other if their centers of mass were within the interaction range of their pair potentials. Fig. 3 summarizes these measurements for a G1 PAMAM dendron-grafted surface which had sustained interactions with the membrane. Most of the interactions occurred between the choline moieties of the lipids and the terminal protonated primary amines of the dendrons, and the phosphate of the phospholipids with the dendron terminal amines. Berényi *et al.*^23^ had reported similar results when studying the effect of G5 PAMAM dendrimers on multilamellar DPPC vesicles. They found that the PAMAM dendrimers tended to favor interactions with the head group region of the lipid bilayers according to Differential Scanning Calorimetry measurements. Other significant interactions which were smaller in magnitude included those between the glycerol groups of the lipids and dendron terminal amines, and the choline and phosphate moieties of the lipids with the amide branches of the dendrons. We report similar trends for the surface grafted with G2 and G3 PAMAM dendrons which maintained sustained interactions with the membrane.

**Fig. 4:**
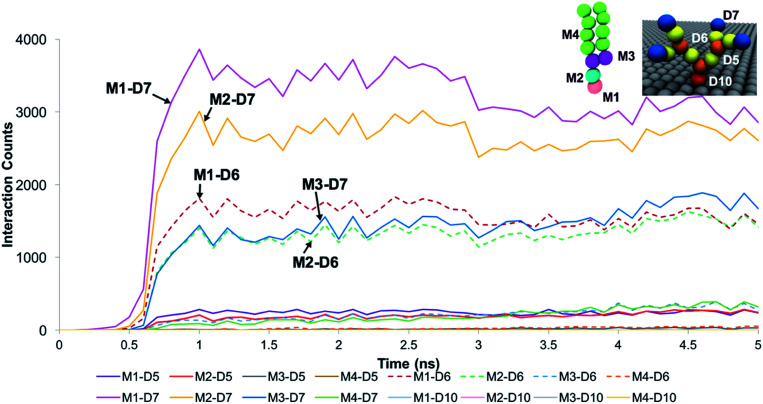
Interaction count measurements between the different bead types of DPPC and the G5 PAMAM dendron when the membrane developed sustained interactions with the dendrons. Choline, phosphate, glycerol and hydrocarbon tail groups of DPPC are labeled respectively as M1, M2, M3 and M4. Terminal amines, amide branches, internal amines and tethering hydrocarbon groups of the PAMAM dendron are labeled respectively as D7, D6, D5 and D10. Measurements for the first 5 ns is shown as the values remain unchanged from 3 ns onwards.

**Fig. 5:**
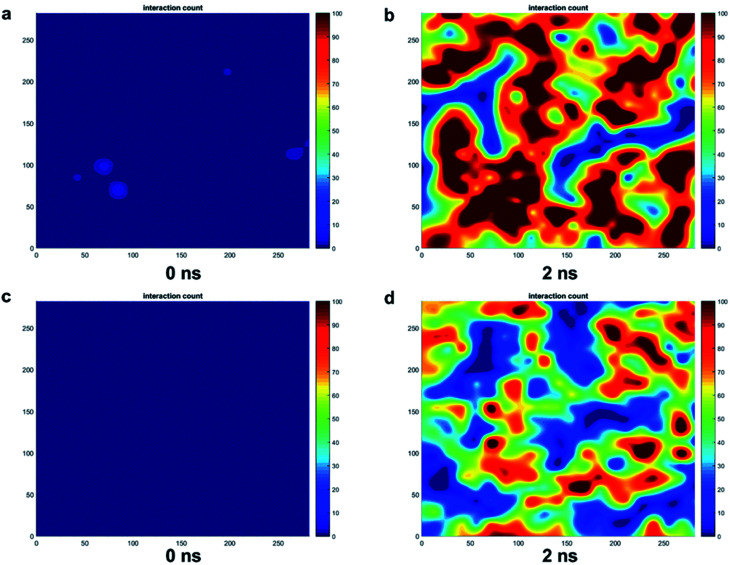
Two-dimensional contour maps of the interaction count for all pairs of bead types between the lipids and (a and b) G1 dendrons at 0 ns and 2 ns and (c and d) G5 dendrons at 0 ns and 2 ns when the membrane developed sustained interactions with the dendrons.

The trends for a G5 PAMAM dendron-grafted surface with sustained interactions with a DPPC membrane were similar to those for G1 with some exceptions (see Fig. 4). Prior to 3 ns, the interactions between the choline groups of the lipids and the amide branches of the dendrons were greater than those between the glycerol moieties of the lipids and the dendron terminal protonated amines. This observation was contrary to the corresponding results for the G1 surface which demonstrated the interactions between the glycerol groups and the terminal amines to remain consistently greater than the choline–amide interactions. In addition, a two-dimensional contour map of the interaction counts for all pairs of bead types between the lipids and the dendrons at 2 ns showed the G1 dendrons to have spatially uniform interactions with the membrane as compared to their G5 counterpart (see Fig. 5). The contour maps illustrate the areas of interaction between the lipid membrane and PAMAM dendrons. They were obtained by evenly dividing the simulation box *x*–*y* plane into 400 sections and counting the numbers of interactions in each section. Even though each G5 dendron had a larger number of beads (in comparison to the G1 dendrons), the number of interactions between the different pairs of beads were consistently lower for the G5 dendrons.

The differences in the interactions of a membrane with the G1 and G5 dendrons can be explained through the following hypothesis. The terminal amines of the PAMAM dendrons all carry positive charges. Due to the repulsion between the terminal amine beads, the beads tend to branch out and away from each other. This endows the PAMAM dendrons with an inherent curvature which mimics a tree-like morphology. The G5 PAMAM dendrons have larger number of dendritic arms and thereby greater curvature as compared to the G1 dendrons (shown in Fig. 1). For a surface grafted with multiple dendrons, the dendrons will adopt conformations which minimize the electrostatic repulsion and maximize their conformational entropy. Hence, only the groups close to the upper curved periphery of each dendron is most likely to interact with the lipid membrane. The remaining groups, including some terminal amines, are concealed away from the membrane. The terminal amines at the top of the dendrons are organized sufficiently far from each other such that the lipids can penetrate the dendrons. As a result, the choline groups are within interaction range of the amide branches. Attraction between oppositely charged moieties in the dendrons and the membrane may result in the terminal protonated amines becoming more closely spaced. This would increase the interactions between the different moieties of the lipid head groups (such as glycerol) and the terminal protonated amines. Our hypothesis is supported by an earlier investigation by Roy *et al.*^57^ on higher generation PAMAM dendrimers. The study showed the PAMAM dendrimers to be less active when interacting with liposomes due to their relatively smaller concentration of total end groups.

For systems where the membrane diffused away from the PAMAM dendron-grafted surface to establish sustained interactions with the counter ions, we observed interactions between the lipid and dendrons to occur only at the beginning of the transient phase (see Fig. SI2†). After the initial interactions between the choline and phosphate groups with the terminal protonated amines, the membrane moved away until it was outside the interaction range from the dendrons. We examined the role of the electrostatic and van der Waals interactions on the processes underlying the sustained interaction of the membrane with either the counter ions or the dendrons. We focused on the most significant interactions between the membrane and the dendrons (namely, those between the choline and phosphate moieties with the terminal amines).

We first considered the impact of electrostatic interactions on the process resulting in the sustained interactions of the membrane with the PAMAM G1 and G5 dendrons. The choline and phosphate groups of DPPC carry positive and negative charges, respectively. At neutral pH, the terminal amines of PAMAM are protonated. The coulombic energies between the lipid charged head groups and the PAMAM terminal protonated amines are provided in Fig. SI3.† The positively charged choline and protonated amines strongly repel each other, as demonstrated by measurements of the corresponding electrostatic potential energy (see Fig. SI3†). Whereas the oppositely charged phosphate and terminal protonated amines strongly attract each other. When we combine the effects of electrostatic attraction and repulsion, we find that the electrostatic attraction dominates. Hence, the membrane moved towards the PAMAM dendrons to develop sustained interactions due to the attraction between the oppositely charged phosphate and terminal protonated amines. Based upon the measurements of the electrostatic potentials, the electrostatic repulsion is relatively strong at the early stage of the transient phase but gradually decreases. After a few nanoseconds, the electrostatic attraction between the phosphate and protonated amine moieties begins to dominate.

For the situation when the membrane developed sustained interactions with the counter ions, the repulsive interactions between the choline and protonated amine groups dominated the electrostatic interactions during the initial transient phase. However, the repulsion decreased as the membrane moved away from the dendrons until they were outside the interaction range from each other (see Fig. SI4†).

We examined the impact of the van der Waals interactions between the different pairs of bead types on the processes underlying the different outcomes. For processes leading to sustained interactions between the membrane and the dendrons, the van der Waals interaction energies between the choline and phosphate groups with the terminal amines were attractive and the most significant. For G1 dendrons as shown in Fig. SI5(a),† the strength of the van der Waals potential energy between the phosphate and terminal amine moieties was almost twice as large as those between the choline and terminal amine groups. The difference in these energies was not as significant for G5 dendrons (see Fig. SI5(b)†). The total van der Waals potential energy for the G1 dendron system was approximately ten times larger than that corresponding to G5. This difference was due to the relatively smaller total number of interacting beads in the G5 system. The favorable van der Waals interactions between the lipids and dendrons further drove the membrane towards the dendron-grafted surface, thereby enabling the development of sustained interactions with both the G1 and G5 dendrons. For the scenario where the membrane developed sustained interactions with the counter ions, the beginning of the transient phase was accompanied by relatively weak van der Waals interactions between the membrane and the dendrons (both G1 and G5). Thereafter, the van der Waals interactions reduce to zero as the membrane moved away from the dendrons.

**Fig. 6:**
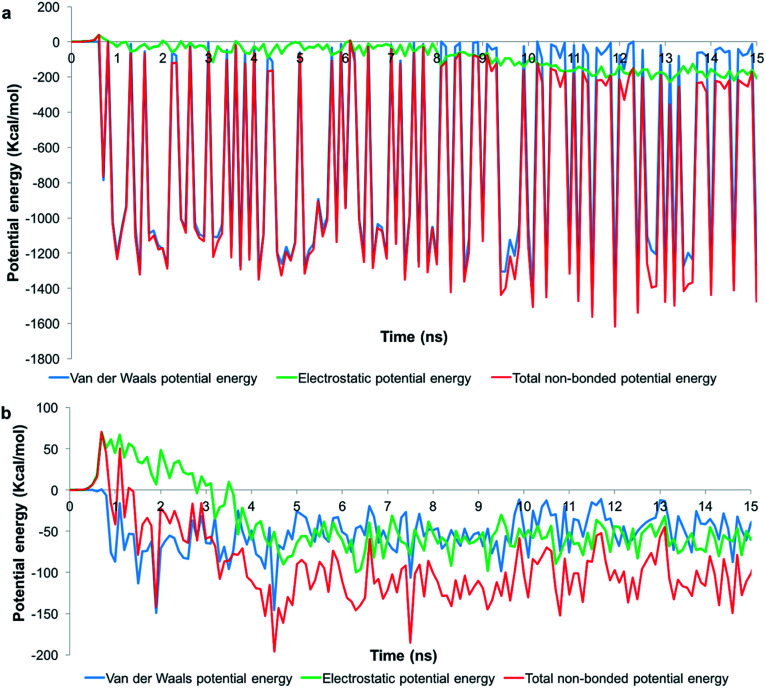
Potential energy measurements when the membrane developed sustained interactions with (a) G1 and (b) G5 PAMAM dendrons. Measurements for the first 15 ns is shown.

To understand the role of the total pair potential on the sequence of processes determining the outcomes for G1 and G5, we combined the electrostatic and van der Waals potential energies between the membrane and the dendrons. Fig. 6 presents the measurements of the first 15 ns for the processes resulting in sustained interactions between the membrane and the dendrons. The results for the entire duration of the simulation are shown in Fig. SI6.† For this scenario, the electrostatic interactions were initially repulsive. As the charged beads were within interaction range from each other, the van der Waals interactions began to increase in strength. During this process, thermal fluctuations could result in the oppositely charged moieties coming within interaction range from each other. This would cause favorable contributions to the electrostatic and van der Waals potential energies. As more oppositely charged moieties begin to interact, both the electrostatic and van der Waals interactions would become increasingly favorable. This sequence of processes would drive the membrane to move towards the dendron-grafted surface and develop sustained interactions with it. It had been reported that only the PAMAM dendrimers with charged terminal groups are capable of causing the formation of holes on cell membranes. Charge neutral dendrimers, by contrast, had no effect on the structure of the membrane.^58^ This finding supports our results in that the interaction between the PAMAM dendrons and the lipid membrane are primarily driven by electrostatic interactions. There are significantly larger fluctuations in the van der Waals potential energy for the G1 dendrons as compared to the G5 dendrons. The difference in the magnitude of the fluctuations is due to a much higher number of interactions between the G1 dendrons with the membrane. The higher density of interactions increases the sensitivity to minor fluctuations at the molecular scale.

For outcomes where the membrane developed sustained interactions with the counter ions (Fig. SI7†), the transient phase began with repulsive electrostatic interactions between the membrane and the dendrons. For the G1 dendrons, the van der Waals interactions were also relatively weak. The dynamics of the molecules did not cause the oppositely charged moieties in the membrane and dendrons to come within interaction range. Hence, the repulsive electrostatic interactions caused the membrane to move away from the dendrons, and eventually develop sustained interactions with the counter ions.

We measured the *z*-component of the average force acting on the DPPC membrane (see Fig. SI8†). For a system which eventually developed sustained interactions with the dendrons, the membrane experienced a relatively strong net force towards the direction of PAMAM dendrons. This was accompanied by a significant increase in the interaction counts. Similarly, a membrane which developed sustained interactions with the counter ions experienced a net positive force (that is, away from the dendron-grafted surface). As there is no force to keep the membrane within interaction range of the dendrons, the inertia of the membrane caused it to move towards the counter ions. Eventually the membrane developed sustained interactions with the counter ions.

For higher generation dendrons (G4 and G5), we observed significantly more number of cases where the membrane developed sustained interactions with the dendrons. We surmise that the architecture of each dendron generation is responsible for this result. Electrostatic repulsion between the protonated terminal amines caused the branches of each dendron to extend and spread away from each other. The dendrons of higher generations have more terminal groups, and thereby more space between the dendron branches. This facilitates penetration by the lipid molecules into the dendron branches. With increasing penetration, more phosphate groups come within interaction range of the terminal amines. Attraction between oppositely charged moieties along with the attractive van der Waals interactions between the different moieties overcomes the effect of thermal fluctuations and drives the membrane towards the dendrons. Our hypothesis is supported by an earlier study.^59^ Huang *et al.* observed similar results when they introduced PAMAM dendrimers on Pt-supported bilayer lipid membranes. The presence of higher generation dendrimers (G4–G7) were observed to induce defects in the lipid membrane. However, the membrane was almost intact when lower generation dendrimers (G1–G3) were added to the solution. This serves to validate our observations.

The dendritic brushes of lower generation dendrons are more closely packed. The tightly packed dendrons facilitate electrostatic interactions between the protonated amines and the charged groups of the lipids. Due to the insufficient space between the terminal groups, the phospholipids are unable to penetrate the dendrons. If the repulsive electrostatic interactions are dominant between the dendrons and the lipids, the thermal fluctuations can induce molecular motion which causes the lipids to move away from the dendrons and escape from their interaction range. Another possibility is that the initial electrostatic repulsion between the lipids and the dendrons are sufficiently strong so as to push the lipids away from the interaction range of the dendrons. In either case, the membrane moves away from the dendrons.

For the situation where the membrane developed sustained interactions with the lower generation dendrons, the initial electrostatic interactions between the lipids and the dendron terminal groups may cause the attractive interactions between the charged moieties to become dominant. With the possible aid of thermal fluctuations, the dendrons assume configurations where the adjacent protonated amines are sufficiently separated from each other so that the lipids can penetrate the dendrons. The sequence of processes thereafter is similar to what is observed for the higher generation dendrons.

### Equilibrium state

The system attained equilibrium as the membrane moved towards the dendrons or the counter ions. The averages with standard deviation of different interaction counts and energies for these two outcomes are provided in Tables SI1 and SI2.† The net charge of the dendrons or the counter ions induced asymmetric stresses across the bilayer. The charged moieties in the lipid head groups were attracted to the oppositely charged groups in the dendrons (or counter ions). The electrostatic attraction lead to changes in the molecular packing of the lipids in the monolayer facing the dendrons (or counter ions). These molecular scale changes result in asymmetric stresses between the lipid monolayers. This stress was released through the development of excess area in one of the monolayers. Since the two leaflets are coupled to form a stable bilayer, the membrane bends to form a bowl-shaped configuration. The bowl-shaped configuration remained stable through the remainder of the simulation. The curved surface of the bowl was observed to be opposite to the dendrons (or counter ions). During this process, the membrane remained structurally stable and did not rupture.

We quantified the deformation of the membrane by measuring its thickness during its interactions with the dendrons (or counter ions). The thickness was determined through the differences in the height of glycerol beads with approximately the same *x*- and *y*-coordinates on the top and bottom membrane leaflets (see Fig. SI9†). The edge of the bowl in a membrane was captured by the dark red circle which indicated that the thickness values of that region was notably higher than the other parts of the membrane. The diameter of the bowls formed for membranes near the dendrons were significantly larger than those formed near the counter ions (see Fig. SI10†). In addition, the diameter of the bowls was observed to typically increase with the PAMAM generation. This can be explained by the excess area induced in the monolayer facing the dendrons or the counter ions. The terminal protonated amines are concentrated in a smaller volume than the counter ions. In addition, the grafting density of dendrons decreases with the generation in order to maintain a consistent surface coverage. The number of G4 and G5 PAMAM dendrons is one ninth of that for G1 PAMAM dendrons, as shown in Table 1. The interaction sites which are at the top of the dendritic brushes are increasingly dispersed from G1 through G5. Hence, dendrons with higher generations will induce larger asymmetric stresses and thereby, higher excess area than counter ions in the same system. A membrane with a larger excess area will form a bowl-shape configuration with a larger diameter.

## Conclusions

We studied the mechanisms underlying the interactions between a DPPC membrane and a PAMAM dendron-grafted surface using the MD technique and a coarse-grained implicit solvent model. The investigation did not consider the presence of membrane proteins, or processes occurring over longer time scales than those captured by the simulations. As a part of the studies, we examined the impact of dendron generation. Our results showed two distinct outcomes: the membrane developing sustained interactions with the dendrons or the counter ions. Higher generation dendrons were found to largely favor sustained interactions with the membrane, in comparison to the lower generation dendrons. This result was due to the electrostatic interactions between the terminal protonated amine groups of the dendrons, their spatial density and interactions with the membrane lipid. Higher generation dendrons were surmised to have greater space between the terminal protonated amine groups, thereby allowing the DPPC molecules to penetrate the dendrons. After the membrane developed sustained interactions with the dendrons or the counter ions, the electrostatic interactions induced asymmetric stresses across the two membrane leaflets resulting in excess area. Hence, the membrane adopted a bowl-shaped morphology whose dimensions depended upon the dendron generations and density of interactions between the membrane and the dendrons or counter ions.

PAMAM dendrimers have been previously reported to disrupt multilamellar DPPC membranes.^23^ The strong binding of charged PAMAM dendrimers onto lipid bilayer membranes can cause the formation of cavity or pores in the membrane.^60^ Sufficiently large asymmetric stresses induced across the bilayer due to interactions with a dendron-grafted surface can rupture the membrane. Therefore, a biological cell membrane interacting with a suitable PAMAM dendron-grafted surface can rupture, thereby killing the cell. Hence, our results can potentially be used to guide experiments for designing of surfaces with selective antifouling properties which can prevent the adsorption of bacteria and other unicellular organisms that have lipid membranes. The grafting density and chemistry of the hyper-branched polyelectrolytes can be varied to optimize the design of the antifouling surface. The dynamical processes underlying the interactions between the lipid membrane and the PAMAM dendron-grafted surface as a function of the dendron generations can guide the characteristics of the surfaces to achieve optimal results for minimizing biofouling.

## Conflicts of interest

There are no conflicts to declare.

## Supplementary Material

RA-008-C8RA03742F-s001
