# Supplementary material for: Mechanisms underlying interactions between PAMAM dendron-grafted surfaces with DPPC membranes
Source: RSC Adv. 2018 Jul 11;8(44):24982–92. doi: 10.1039/c8ra03742f (PMC9082323; doi:10.1039/c8ra03742f)
Supplement: RA-008-C8RA03742F-s001 [file RA-008-C8RA03742F-s001.pdf]

This journal is © The Royal Society of Chemistry 2018

Supporting Information.

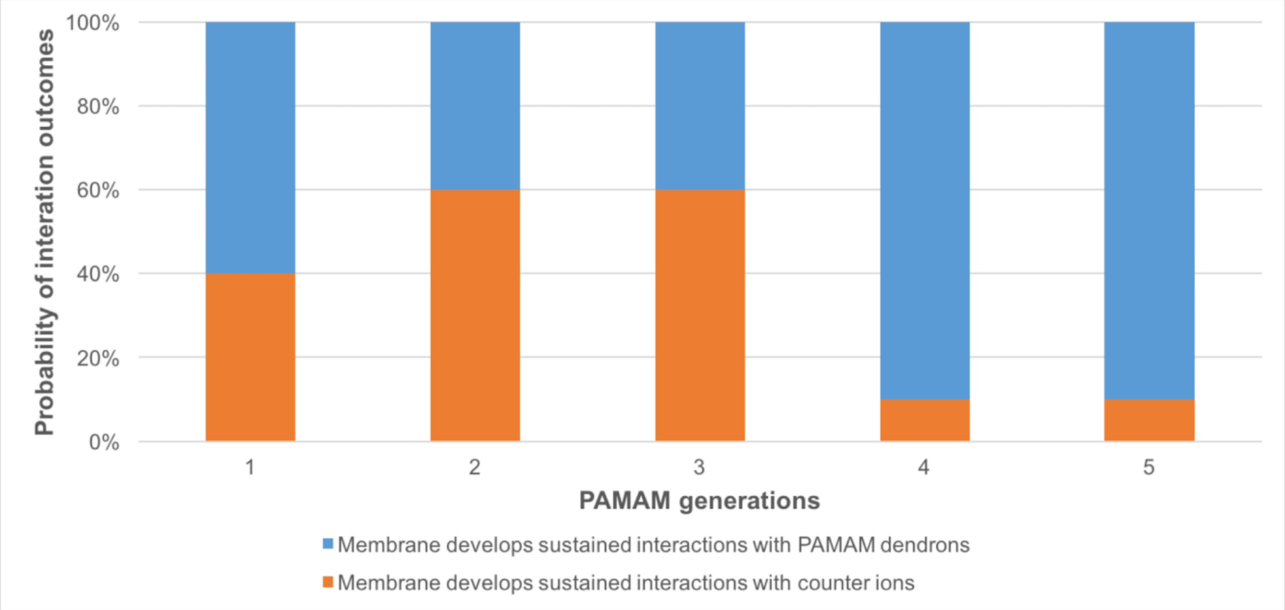

Figure SI1. Probability of interaction outcomes between lipid membrane and PAMAM dendrons for different dendron generations.

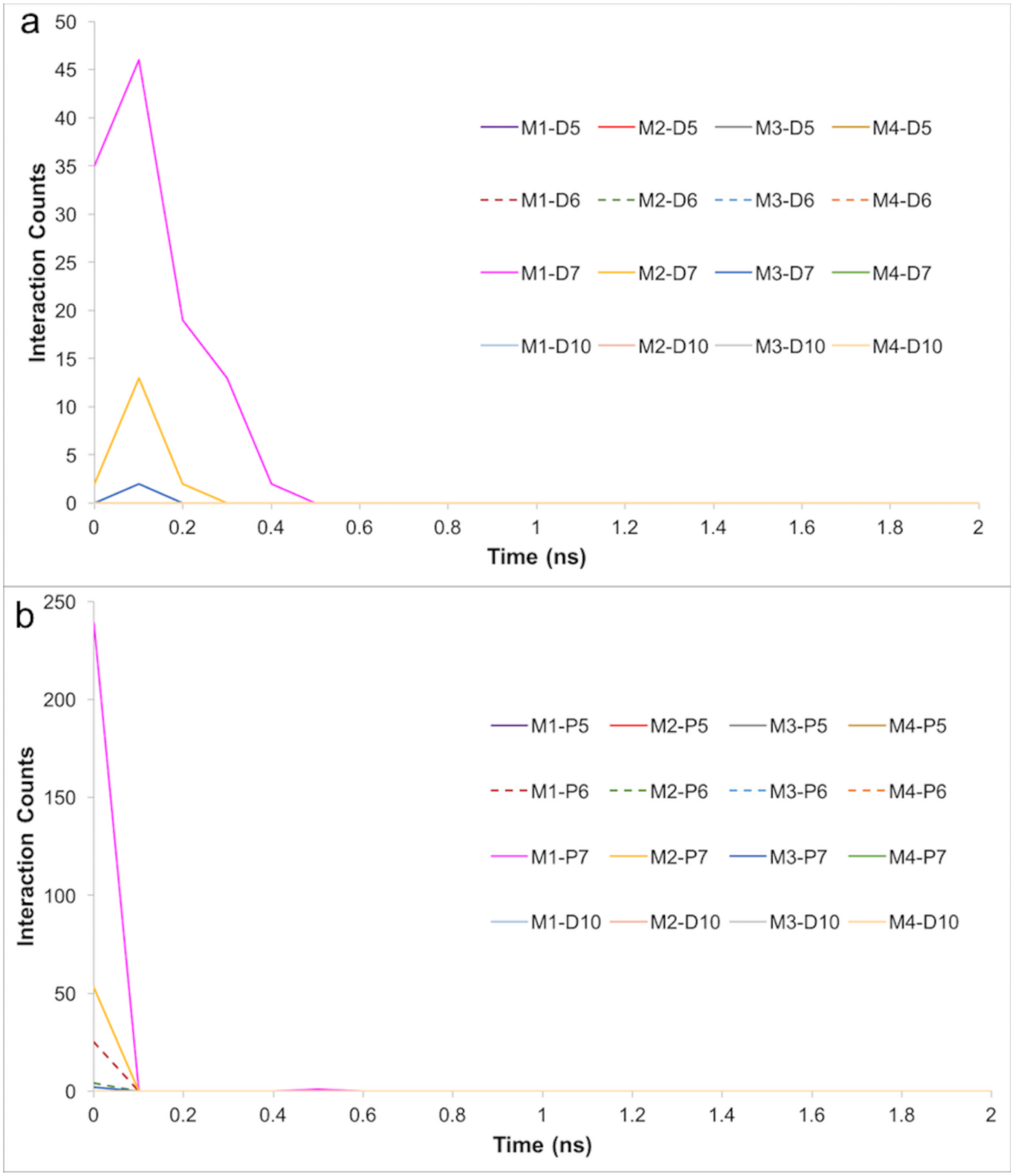

Figure SI2. Interaction count measurements when membrane develops sustained interactions with counter ions for systems with (a) G1 and (b) G5 PAMAM dendrons.

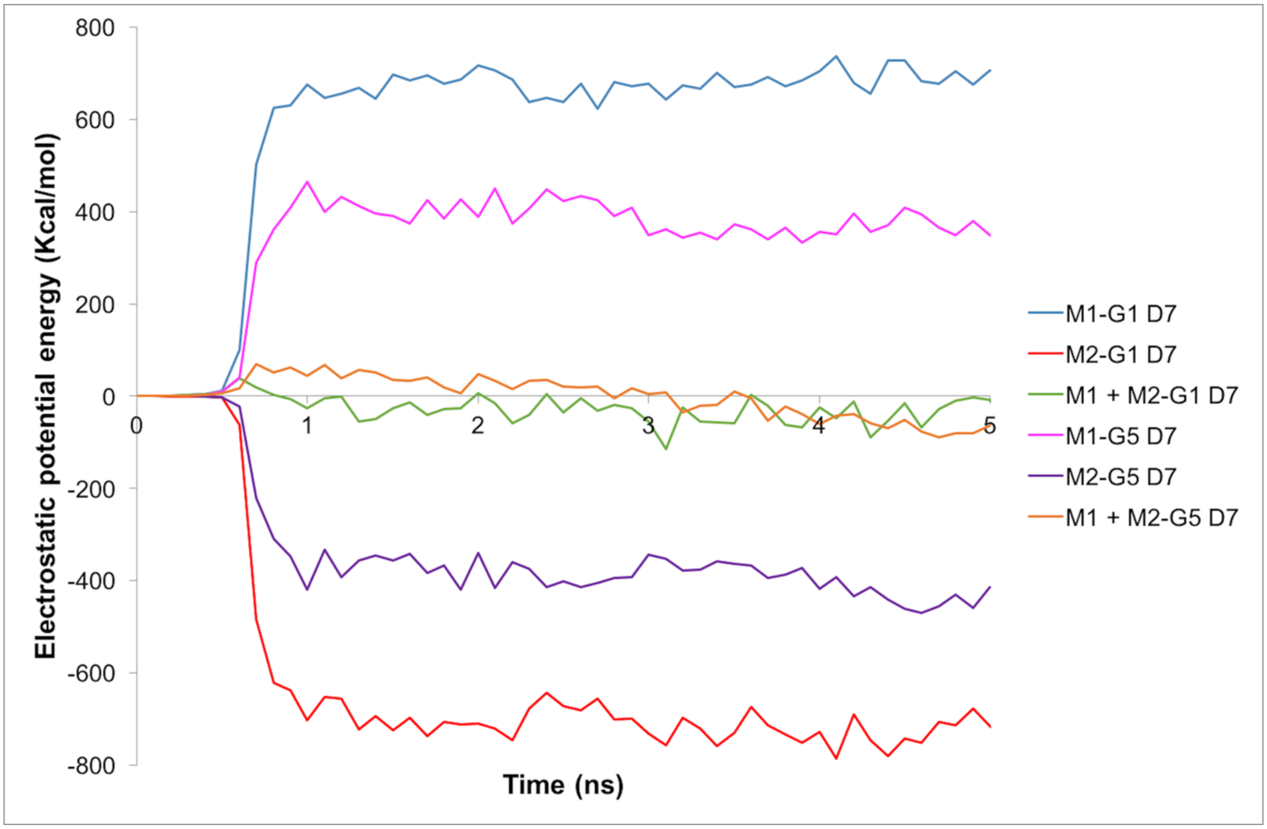

Figure SI3 Electrostatic potential energy measurements for DPPC and G1 and G5 PAMAM dendrons when the membrane developed sustained interactions with the dendrons. Choline, phosphate of DPPC and terminal amines of the PAMAM dendron are labeled respectively as M1, M2, and D7. Measurements for the first 5 ns is shown as the values remain unchanged from 4 ns onwards.

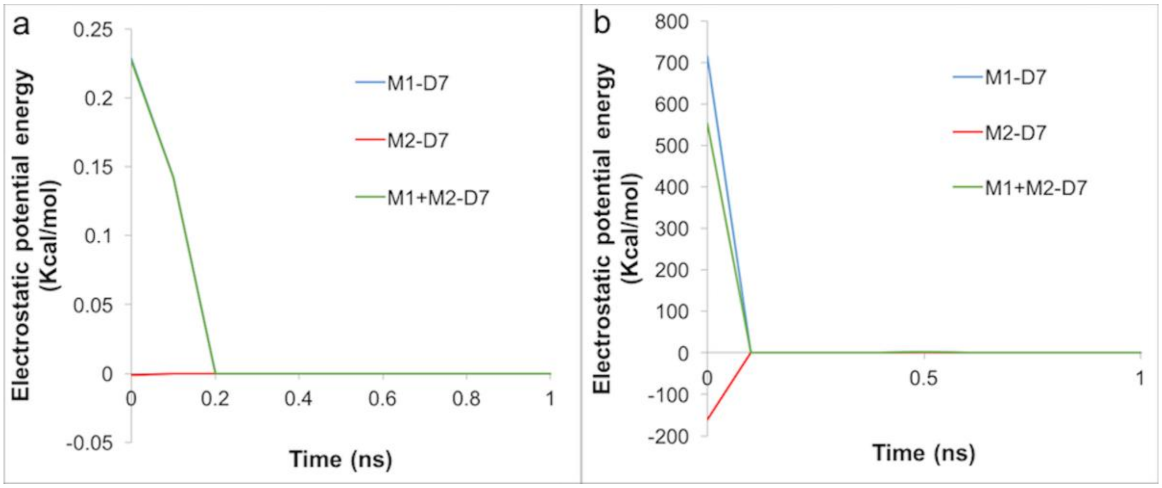

Figure SI4. Electrostatic potential energy measurements when membrane develop sustained interactions with counter ions in system with (a) G1 and (b) G5 PAMAM dendron.

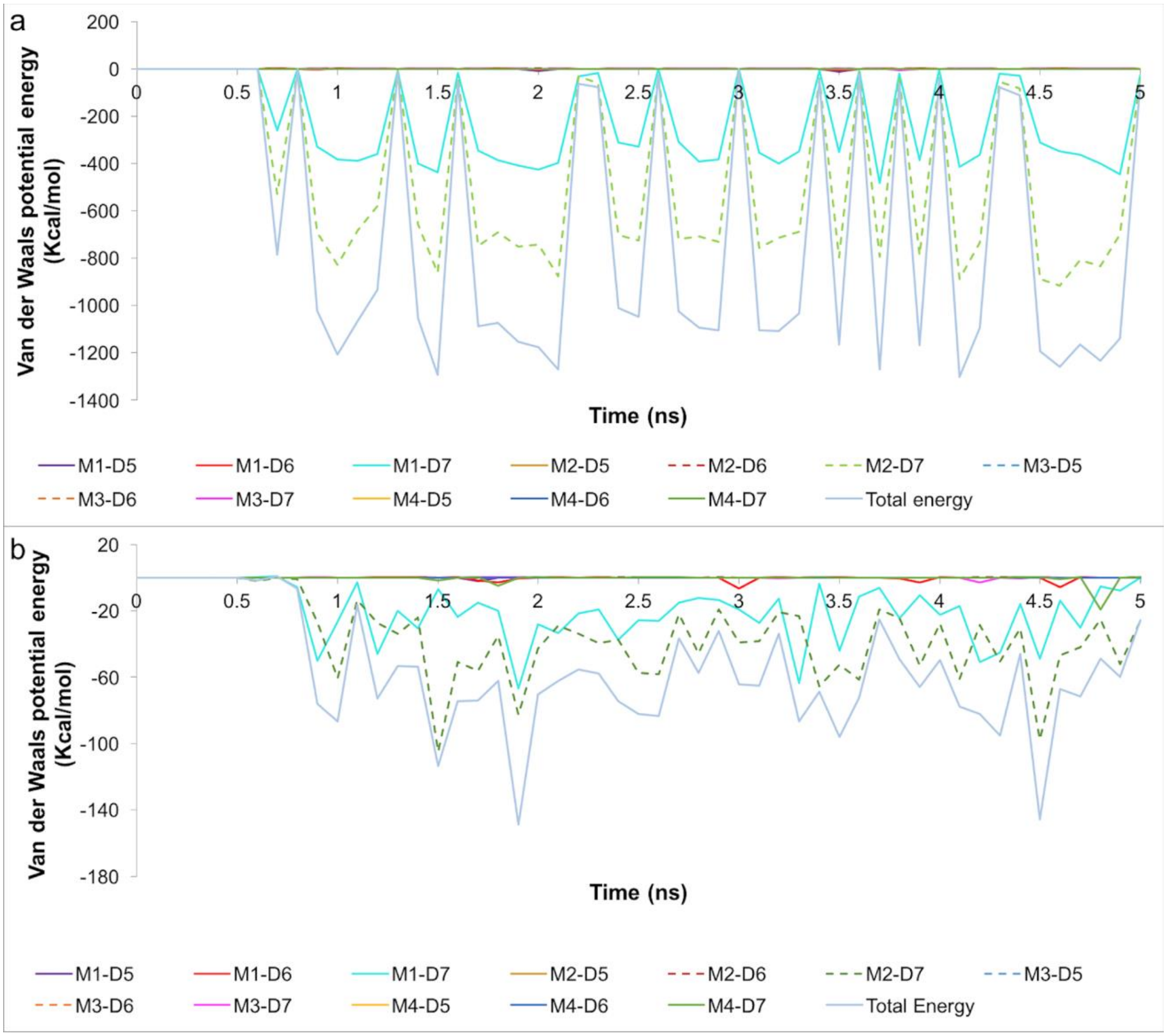

Figure SI5. Van der Waals potential energy measurements when membrane develops sustained interactions with (a) G1and (b) G5 PAMAM dendron.

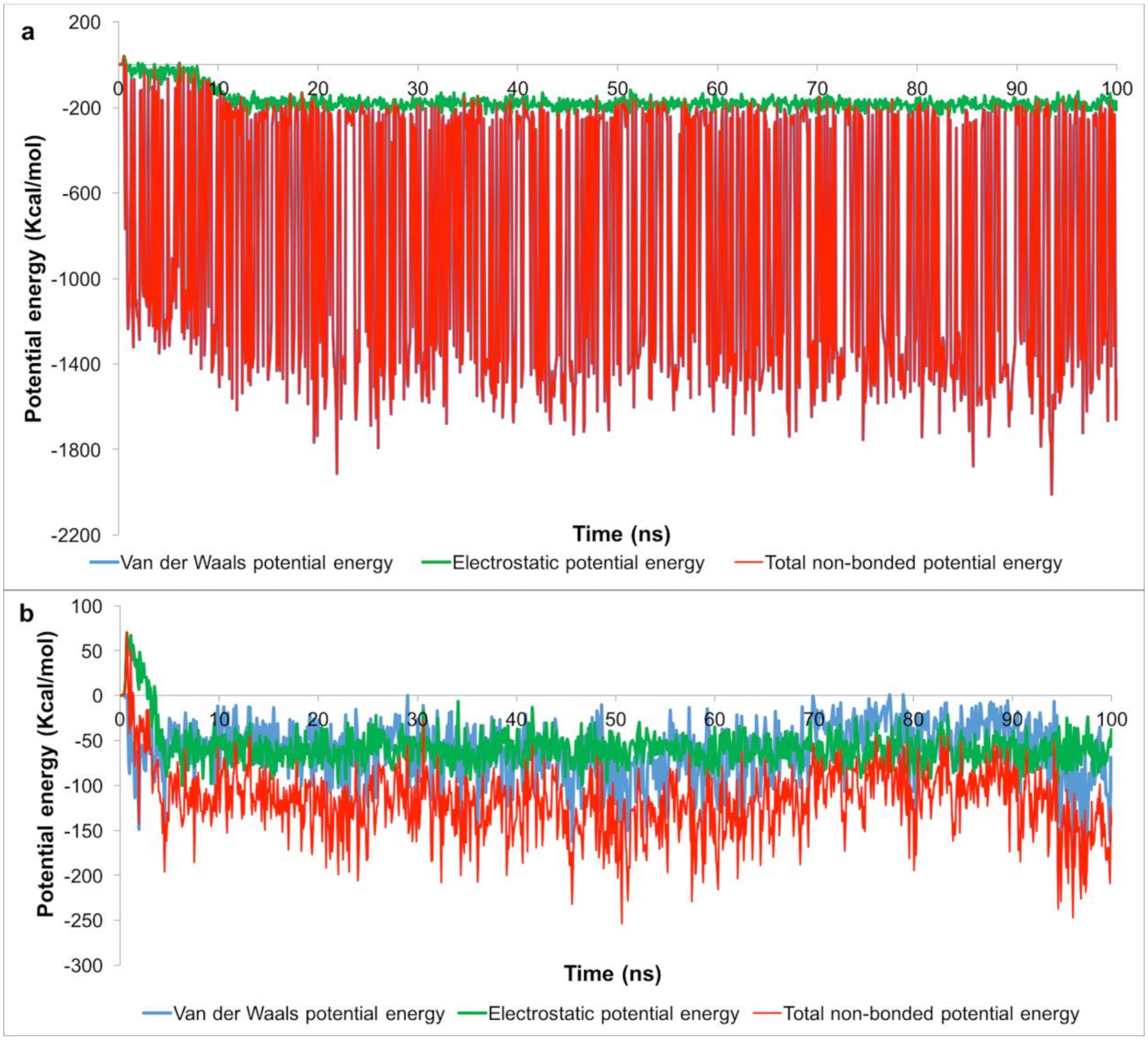

Figure SI6. Potential energy measurements when the membrane developed sustained interactions with (a) G1 and (b) G5 PAMAM dendrons.

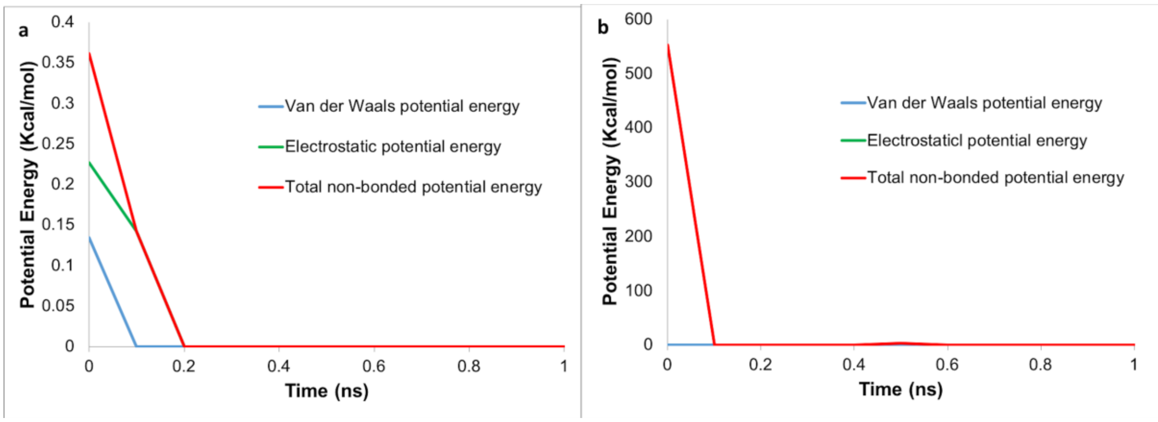

Figure SI7. Potential energy measurements when membrane develops sustained interactions with counter ions in system with (a) G1and (b) G5 PAMAM dendron.

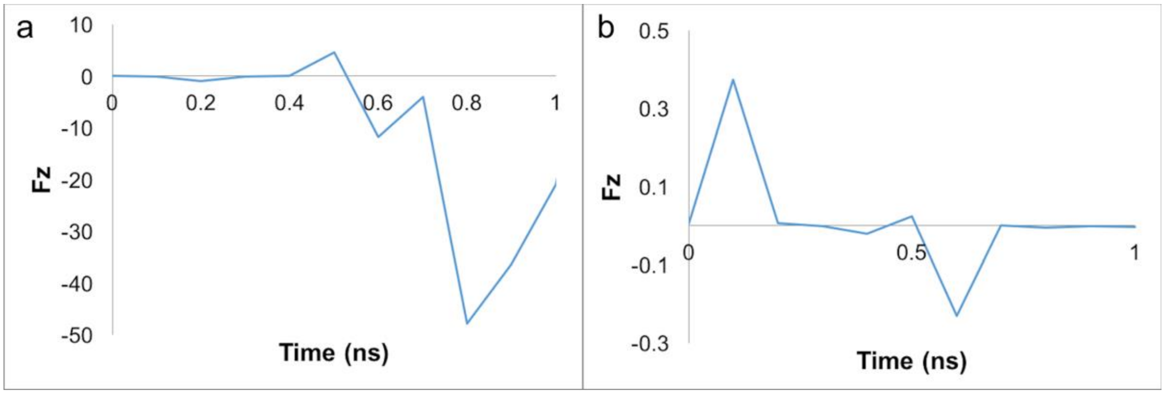

Figure SI8. Z-component average force acting on DPPC membrane when membrane develops sustained interactions with (a) G1 PAMAM dendron and (b) counter ions in system with G1 PAMAM dendron.

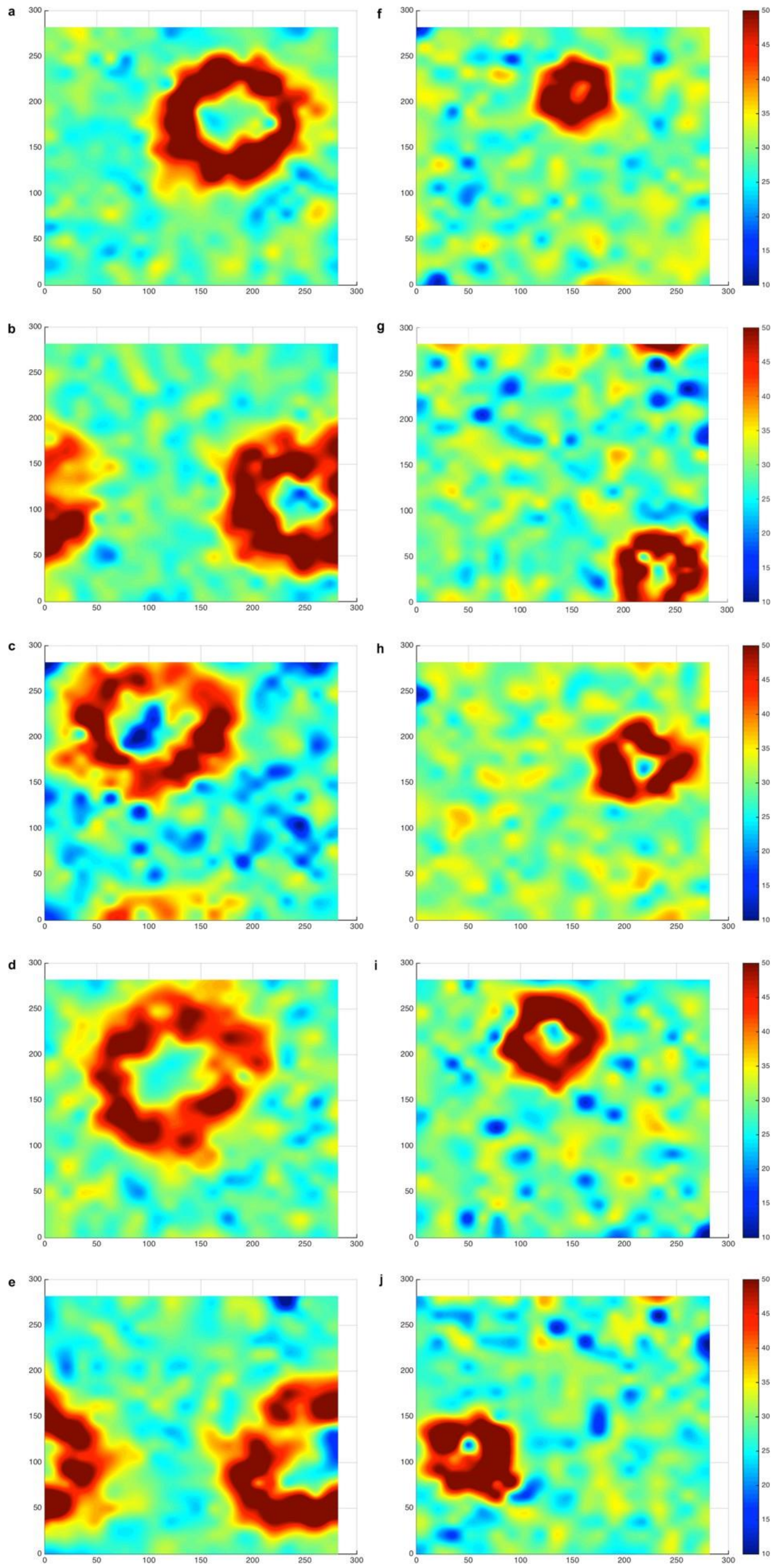
a
f
b
g
c
h
d
i
e
j

Figure SI9. Two-dimensional contour maps of the membrane thickness for a membrane interacting with (a-e) G1 through G5 PAMAM dendrons and (f-j) the corresponding counter ions.

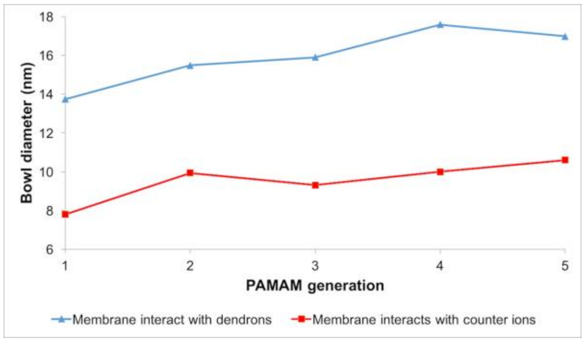

Figure SI10. Bowl size measurements when membrane interacts with dendrons and counter ions of different dendron generations.

Table SI1. Average and standard error values for the outcomes of G1 and G5 membrane interacting with dendrons for equilibrium state (30 – 100 ns)

		Average	Standard deviation
G1	Interaction Count	16693.95	797.33
	Van der Waals Potential Energy (Kcal/mol)	-675.40	597.22
	Electrostatic Potential Energy (Kcal/mol)	-183.65	22.05
	Total non-bonded Potential Energy (Kcal/mol)	-859.05	598.17
G5	Interaction Count	11596.02	679.79
	Van der Waals Potential Energy (Kcal/mol)	-61.99	37.25
	Electrostatic Potential Energy (Kcal/mol)	-59.84	15.39
	Total non-bonded Potential Energy (Kcal/mol)	-121.83	41.09

Table SI2. Average and standard error values for the outcomes G1 and G5 of membrane interacting with counter ions for equilibrium state (30 – 100 ns)

		Average	Standard deviation
G1	Interaction Count	0.00	0.00
	Van der Waals Potential Energy (Kcal/mol)	0.00	0.00
	Electrostatic Potential Energy (Kcal/mol)	0.00	0.00
	Total non-bonded Potential Energy (Kcal/mol)	0.00	0.00
G5	Interaction Count	0.00	0.00
	Van der Waals Potential Energy (Kcal/mol)	0.00	0.00
	Electrostatic Potential Energy (Kcal/mol)	0.00	0.00
	Total non-bonded Potential Energy (Kcal/mol)	0.00	0.00
